# Low-cost optofluidic add-on enables rapid selective plane illumination microscopy of *C. elegans* with a conventional wide-field microscope

**DOI:** 10.1117/1.JBO.26.12.126501

**Published:** 2021-12-10

**Authors:** Mehran Behrouzi, Khaled Youssef, Pouya Rezai, Nima Tabatabaei

**Affiliations:** York University, Lassonde School of Engineering, Department of Mechanical Engineering, Toronto, Canada

**Keywords:** selective plane illumination microscopy, light-sheet fluorescence microscopy, *C. elegans*, microfluidics, optofluidic, organism-on-a-chip

## Abstract

**Significance**: Selective plane illumination microscopy (SPIM) is an emerging fluorescent imaging technique suitable for noninvasive volumetric imaging of *C. elegans*. These promising microscopy systems, however, are scarce in academic and research institutions due to their high cost and technical complexities. Simple and low-cost solutions that enable conversion of commonplace wide-field microscopes to rapid SPIM platforms promote widespread adoption of SPIM by biologist for studying neuronal expressions of *C. elegans*.

**Aim**: We sought to develop a simple and low-cost optofluidic add-on device that enables rapid and immobilization-free volumetric SPIM imaging of *C. elegans* with conventional fluorescent microscopes.

**Approach**: A polydimethylsiloxane (PDMS)-based device with integrated optical and fluidic elements was developed as a low-cost and miniaturized SPIM add-on for the conventional wide-field microscope. The developed optofluidic chip contained an integrated PDMS cylindrical lens for on-chip generation of the light-sheet across a microchannel. Cross-sectional SPIM images of *C. elegans* were continuously acquired by the native objective of microscope as worms flowed in an L-shape microchannel and through the light sheet.

**Results**: On-chip SPIM imaging of *C. elegans* strains demonstrated possibility of visualizing the entire neuronal system in few seconds at single-neuron resolution, with high contrast and without worm immobilization. Volumetric visualization of neuronal system from the acquired cross-sectional two-dimensional images is also demonstrated, enabling the standard microscope to acquire three-dimensional fluorescent images of *C. elegans*. The full-width at half-maximum width of the point spread function was measured as 1.1 and 2.4  μm in the lateral and axial directions, respectively.

**Conclusion**: The developed low-cost optofluidic device is capable of continuous SPIM imaging of *C. elegans* model organism with a conventional fluorescent microscope, at high speed, and with single neuron resolution.

## 1 Introduction

*Caenorhabditis elegans (C. elegans)* is a widely used model organism for studying molecular, cellular, and behavioral mechanisms underlying human diseases.^1^^,^^2^ The success of such investigations frequently relies on availability of imaging platforms that can visualize *C. elegans* neuronal activities at high spatial and temporal resolutions. Wide-field (WF) fluorescent microscopy is commonplace in biology but suffers from poor image contrast due to presence of out-of-focus/background fluorescence. Confocal fluorescent microscopy (CM), on the other hand, offers high-contrast images of *C. elegans* neuronal activities but, similar to WF, is prone to photodamage and photobleaching.^3^^,^^4^

To overcome the above limitations, light-sheet microscopy techniques, also known as selective plane illumination microscopy (SPIM), have been proposed.^5^^–^^8^ SPIM systems illuminate the sample only in the focal plane of the detection objective to enable acquisition of high contrast images with minimal photodamage. Enhancement in imaging speed, compared with CM, is another key advantage of SPIM as the image is acquired in a widefield manner.^9^ To date, several variants of SPIM have been developed and tailored for imaging biological samples, and in particular for imaging *C. elegans*.^10^^–^^13^ These standalone systems, however, are normally complex and costly because: (1) they require two dedicated illumination and detection objectives; (2) they require controlled/motorized translation of immobilized *C. elegans* through the light-sheet for three-dimensional (3D) image acquisition. These limitations have effectively hindered widespread adoption of SPIM by biologists.

In an attempt to make SPIM more accessible, a collection of recent works has focused on making SPIM compatible with the commonplace WF microscopes.^14^^–^^19^ For example, replacing the epi-illumination of the host conventional microscope with light-sheet illumination has shown to enable acquisition of light-sheet fluorescent images with high signal-to-background ratio.^14^^,^^17^ Another example is the work of Hsieh et al.^16^ in which a volume holographic optical element was developed to create a light-sheet for high contrast imaging of *C. elegans* with the conventional WF microscope. These promising works, however, were quite complex and slow as *C. elegans* had to be immobilized in gel-based media and translated through the light sheet via precision motorized components. In another approach, on-chip platforms (e.g., microfluidics devices) were integrated into conventional SPIM systems to enable fast fluorescent imaging of *C. elegans*.^20^ While this approach significantly enhanced the imaging speed by acquiring images of *C. elegans* as they flowed through the light-sheet, the proposed solution was costly because it utilized a standalone SPIM system. As such, development of low-cost, yet rapid, innovations suitable for SPIM imaging of *C. elegans* are yet to be reported.

In this work, we report on the design, development, and validation of a polydimethylsiloxane (PDMS)-based optofluidic chip for simple, low-cost, and rapid SPIM imaging of *C. elegans*. Unlike previous works that required either dedicated/costly SPIM systems or sample immobilization in gel-based media, the developed innovation is compatible with a conventional WF microscope and performs SPIM imaging as *C. elegans* are pumped through a microchannel. To do so, a cylindrical PDMS lens was integrated into the developed optofluidic chip to generate a light sheet across the cross-section of a microchannel. This approached enabled us to use the native objective lens of a conventional WF microscope to continuously acquire SPIM images of cross sections of worms flowing through the stationary light sheet. The flow-based translation of *C. elegans* through the light sheet is specifically valuable as it eliminates the complexities associated with immobilizing *C. elegans* in gel-based media. The results of our feasibility study demonstrate the possibility of continuous SPIM imaging of the cross sections of *C. elegans* to visualize single neurons and the entire neuronal system with high contrast and at high speed (∼2  s per worm). We anticipate this miniaturized and low-cost platform to enable widespread adoption of SPIM in biology laboratories for rapid and high-contrast volumetric imaging of *C. elegans*.

## 2 Experimental Methodology

### 2.1 Overview of the Low-Cost Optofluidic SPIM Platform

**Fig. 1:**
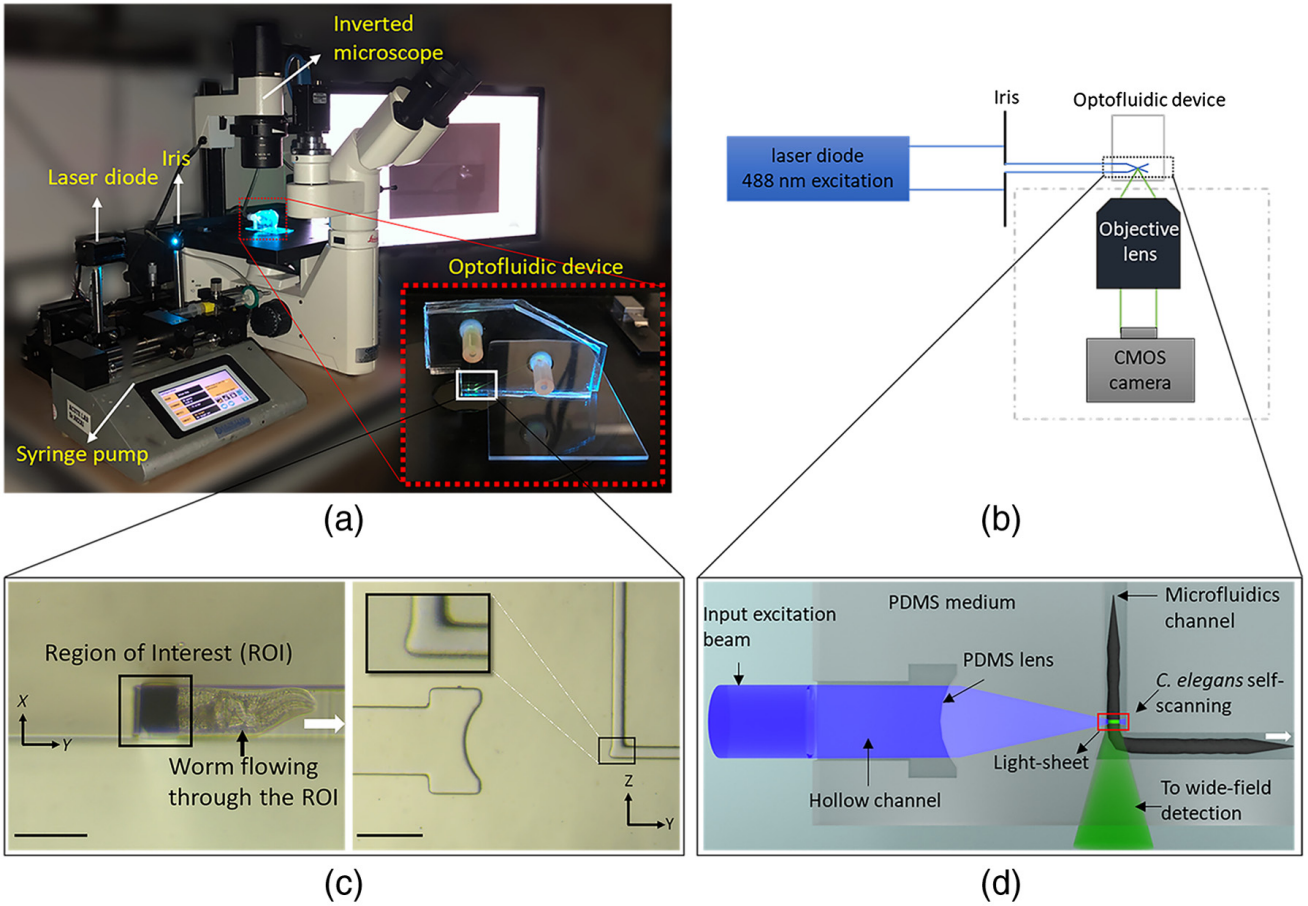
Illustration of the developed low-cost optofluidic SPIM platform. (a) The experimental setup included a free-space and low-cost laser diode and an iris that were used to generate the input excitation beam of the optofluidic device. *C. elegans* were loaded into the device using a syringe pump. The optofluidic add-on device on top of the inverted wide-field fluorescent microscope is shown in the inset of (a). (b) Schematic of the platform, not drawn to scale. The generated light-sheet replaced the epi-illumination of the host microscope. The emitted fluorescent was collected by the native objective lens of the microscope and recorded using a CMOS camera. (c) Side-view (left image, scale bar 100  μm) and top-view (right image, scale bar 200  μm) bright-field images of the optofluidic device taken by the microscope. A worm flowing through the ROI is shown in the left image; white arrow shows the direction of flow. The magnified inset of the right image shows the inverse tapered area of the channel designed to accommodate the numerical aperture of the detection lens. (d) Working principle of the optofluidic device. Collimated input beam was focused using the integrated PDMS lens and light-sheet was generated inside the microfluidics channel. The cross-section of the worm was continuously imaged as worm traveled through the light-sheet. Optically sectioned images were acquired through the smooth PDMS wall at the bottom of the optofluidic add-on device.

The experimental setup of the low-cost optofluidic SPIM platform is shown in Fig. 1(a). Briefly, the developed optofluidic add-on device was placed on the imaging platform of a conventional WF microscope for light-sheet imaging [inset in Fig. 1(a)]. This device enabled volumetric SPIM imaging of *C. elegans* without sample immobilization and mechanical scanning. The input beam to the add-on device was provided by a free-space and low-cost laser diode source and adjusted by an iris (λ: 488 nm, 3 mW, OXlasers A-B60F, China; Fig. 1). The integrated PDMS cylindrical lens of the add-on device created the illumination light sheet across a microchannel. The emitted fluorescent light from the sample was collected with the native air objective lens of the inverted wide-field microscope (N PLAN 20X/0.35NA; DM IL LED microscope; Leica, Wetzlar, Germany). Optically sectioned images were recorded with a 5-Mpixels CMOS camera with a pitch size of 0.31  μm (GS3-U3-51S5M-C, Point Grey, Canada).

To enable flow-based imaging of samples, a syringe pump (legato 180, KD scientific) was used to pass the *C. elegans* through the stationary light-sheet at a reasonably constant flow rate. For detailed visualization of the neuronal system, the flow velocity was chosen as $∼90\;\mu m\cdot s^{-1}$. At this velocity, an entire worm was scanned in ∼10  s with the camera operating at 150 fps. For fast imaging of *C. elegans*, considering that our SPIM system images the cross-section of the worm, the active area of the camera was reduced to only cover the required field of view. This, in return, enabled us to use higher frame rates up to 300 fps, corresponding to 100  μm×100  μm imaging area/region of interest (ROI), Fig. 1(c) (left). In this configuration, the flow velocity was chosen as $∼450\;\mu m\cdot s^{-1}$. In both imaging speed cases, at least one frame was acquired per a sample movement equal to the light-sheet beam waist to ensure Nyquist sampling. The volume viewer plugin of Fiji^21^ was used to generate 3D volumetric image of *C. elegans* from the acquired two-dimensional (2D) cross-sectional images.

#### 2.1.1 Mechanical design

The top- and side-view microscopic images of the optofluidic device and its schematical working principle are shown in Figs. 1(c) and 1(d), respectively. The device consisted of two parts: the microfluidic channel for *C. elegans* transport, and the integrated PDMS lens for light-sheet generation. For *C. elegans* transportation, the microfluidic channel was designed in an L-shape form to minimize the aberrations caused by the presence of the worm in the optical path of the detection objective lens. Microchannel cross-sectional dimensions were ∼50  μm×65  μm to enable smooth passing of a single adult worm (∼50-μm diameter) through the imaging plane with minimal lateral movement. The bottom section of the L-shaped channel was designed in an inverse taper shape (∼20  deg) to accommodate the 0.35 numerical aperture of the objective lens, Figs. 1(c) (right) and 1(d). In such an arrangement, the emitted fluorescent light from the sample passed through a small section of the channel, the PDMS device wall thickness, and air before reaching the objective lens. To minimize the degradation of image quality, the PDMS device was cut carefully to obtain an ∼400-μm-thick PDMS wall. To remove the surface roughness of the cut PDMS–air interface, an additional step was taken to make the rough facet as optically flat as possible; the optofluidic device was fabricated using standard photo- and soft-lithography techniques (details in the Supplemental Material).^22^

#### 2.1.2 Optical design

**Fig. 2:**
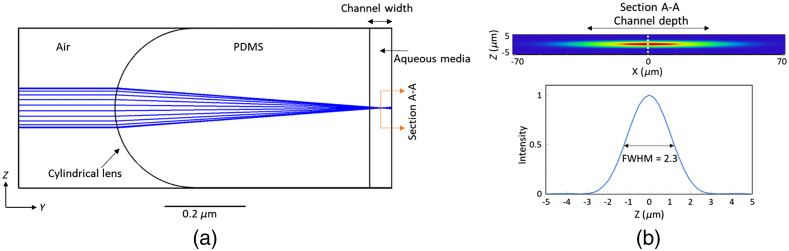
Optical simulation using Zemax OpticStudio^®^. (a) Lateral view of the ray tracing simulation used for determining the geometrical position, radii of curvature, and aperture size. The coupled beam is focused owing to the refractive index mismatch of air and PDMS, and consequently the light-sheet is generated within the width of the channel. (b) Cross-sectional light intensity profile of the light-sheet at the center of the channel at the focus (section A-A in a), simulated using physical optics propagation tool. The FWHM measurement for the light-sheet thickness using intensity distribution along z axis at x=0 is also shown.

The illumination part of the optofluidic device for light-sheet generation was designed and optimized using Zemax OpticStudio® (version 20.1.2). To do so, an air microcavity within the PDMS chip was simulated, Fig. 2. Given the refractive index mismatch of air and PDMS (1 versus ∼1.42),^23^ the cavity’s distal end was shaped in form of a convex cylindrical lens to enable the on-chip generation of the light sheet across the cross section of the microfluidic channel.^24^ The intrinsic manufacturing precision of monolithic photo- and soft-lithography enabled proper alignment of the light-sheet with respect to the microfluidics channel during the fabrication stage. Given the ∼50-μm diameter of *C. elegans*, at least 50-μm light-sheet length (defined as twice the Rayleigh length) was desired to uniformly illuminate the worm along its cross-section. To achieve this light-sheet length with the 488-nm excitation laser, a light-sheet thickness of ∼2.3  μm (i.e., full-width at half-maximum, FWHM, at the focus) was required based on the Gaussian beam optics.^25^ Zemax simulations determined that a 200-μm radius of curvature of the air–PDMS interface of microcavity can satisfy the required light-sheet specifications. Furthermore, the Zemax ray optics module and the native merit function were used to optimize the distance between the curved interface and the microfluidics channel to locate the beam focus at the center of the microfluidic channel, Fig. 2(a). Zemax physical optics tool determined an optimized uniform light-sheet illumination across the microchannel with beam thickness of 2.3  μm at the channel center from an input Gaussian beam with $1/e^{2}$ diameter of 90  μm, Fig. 2(b). Simulations also demonstrated that light sheet thickness can be changed between 2 and 3  μm by changing the input beam diameter in the range of ∼60 to ∼120  μm which can be beneficial for tuning the resolution based on the different stages of *C. elegans* under study.

### 2.2 Optical Characterization

A beam profiler camera (WinCamD-UHR–½ in. CMOS Beam Profiler; DataRay) was used to characterize the output beam of the laser. Optical characterization of the optofluidic device was carried out with a Rhodamine fluorescent dye (Rhodamine 110 chloride, Sigma-Aldrich). To measure the thickness of light-sheet, the add-on device was placed on the inverted microscope in top-view orientation [Fig. 1(c), right image], Rhodamine solution was pumped into the microchannel, and the fluorescent expression was imaged. Imaging resolution of the system was determined from SPIM images of 500-nm fluorescent beads (FP-0556-2, fluorescent Nile red particles, Spherotech). The beads were diluted in aqueous solution and continuously imaged as they flowed through the stationary light-sheet. The experimental lateral and axial resolutions of the system were assessed by analyzing the acquired consecutive frames of passing beads.

### 2.3 *C. elegans* Preparation

NW1229 [expressing green fluorescent protein (GFP) pan-neuronally; F25B3.3::GFP+dpy-20(+)] and BZ555 (expressing GFP in dopaminergic neurons (DNs); dat-1p::GFP) *C. elegans* strains were purchased from the *Caenorhabditis* Genetics Center (University of Minnesota). All worms were maintained at room temperature on freshly prepared nematode growth media plates seeded with *Escherichia coli* (*E. coli*) strain OP50 as a food source.^26^ All worm maintenance procedures were performed under biosafety number 02-19 issued by York University’s Biosafety Committee to PR. All experiments were performed with age-synchronized, well-fed gravid hermaphrodite adults (∼60  h), using the conventional alkaline hypochlorite treatment method (details in the Supplemental Material).^27^

## 3 Results and Discussion

### 3.1 Optical Characterization of the Add-On Optofluidic Device

**Fig. 3:**
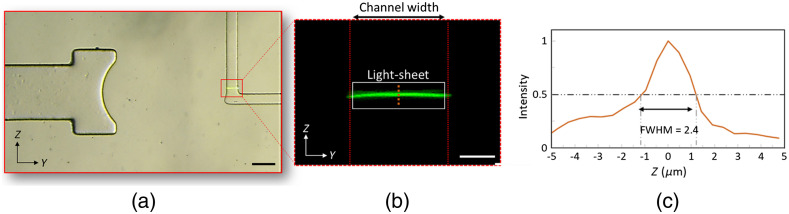
Optical characterization of the add-on optofluidic device. (a) Overlaid bright-field and light-sheet images of the device filled with rhodamine fluorescent dye (100  μm scale bar). (b) Magnified GFP image of the L-shaped microchannel showing the generated light-sheet taken by the green filter (525±50  nm band pass) of the microscope (20  μm scale bar). (c) A 2.4-μm FWHM was achieved for the light-sheet thickness at the focus, at the middle of the channel along the vertical dashed line shown in (b).

Once the device was fabricated, optical characterization tests were carried out to measure the light-sheet thickness and the experimental resolution of the imaging system. Figures 3(a) and 3(b) show the bright-field and the fluorescent images of rhodamine solution, respectively. From the intensity measurements in Fig. 3(c), an FWHM of 2.4  μm was measured as the light-sheet thickness at the focus (i.e., at the middle of the channel). The small deviation of the measured value from the simulated one (2.4 versus 2.3  μm) can be due to measurement errors.

**Fig. 4:**
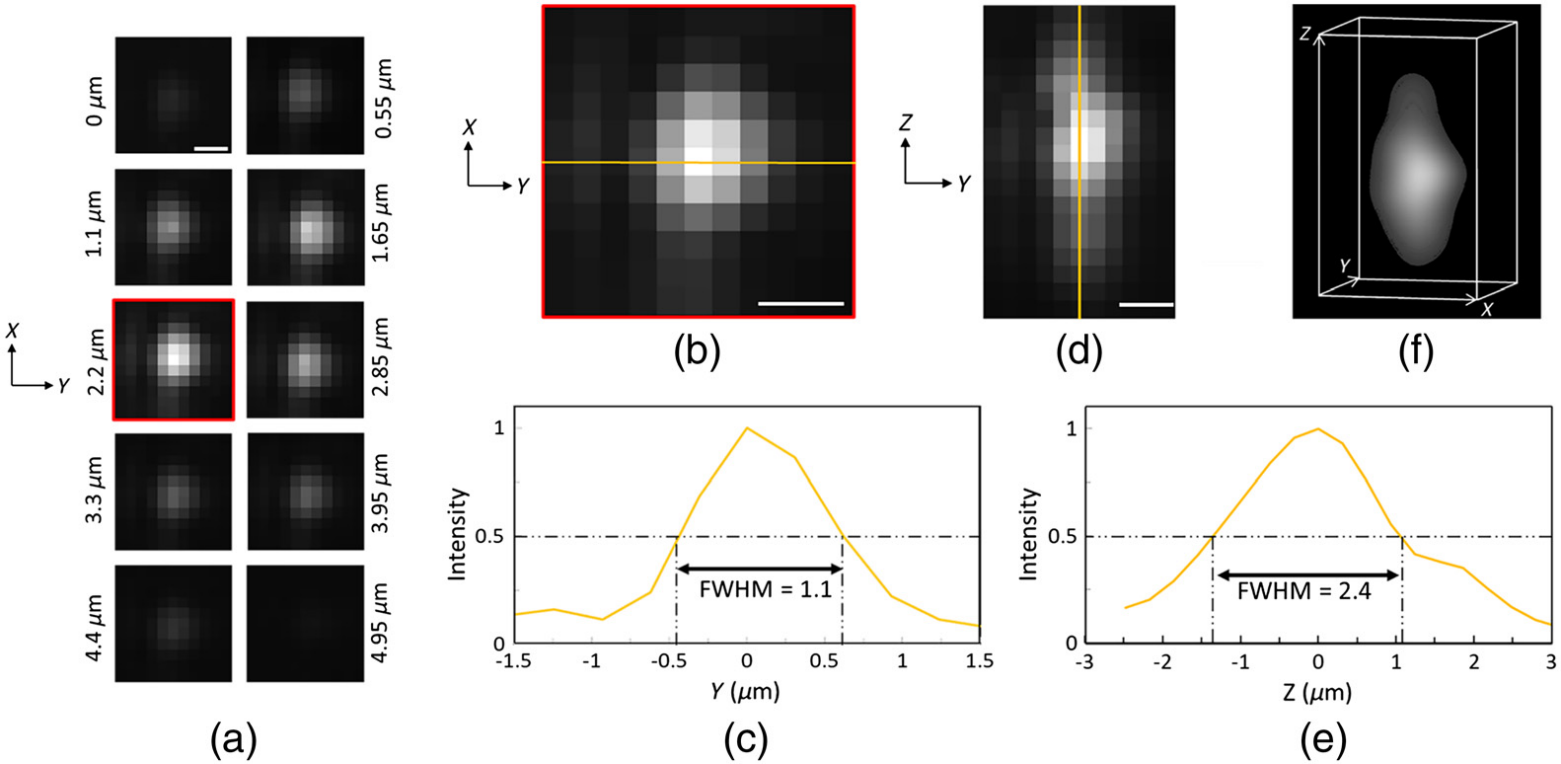
Resolution measurement of the optofluidic SPIM platform. (a) Acquired images of a 500-nm fluorescent bead passing through the light-sheet. Images are shown at different time points corresponding to 0.55-μm axial spacing. (b) Magnified image of the central plane of the bead in (a). (c) The intensity profile along the line in (b) showing a lateral FWHM of 1.1  μm. (d) Axial reconstruction of the stack of images in (a). (e) The axial intensity profile along the line shown in (d). (f) 3D experimental PSF acquired with volumetric rendering of the stack of images in (a). Scale bars in panels (a), (b), and (d) correspond to 1  μm.

Figure 4 shows the imaging results from the 500-nm fluorescent beads (aka point source) captured with the 20×/0.35NA objective lens of the WF microscope. A sequence of images from a bead passing through the light sheet is shown in Fig. 4(a). The axial step size of image sequence was measured by particle trajectory as 0.55  μm. Figure 4(b) shows the cross-sectional image of the resolved bead at the center of the light sheet; the normalized intensity profile across the center of the image [Fig. 4(c)] suggests a lateral FWHM of 1.1  μm. The measured FWHM is 24% inferior to the theoretical one (0.83  μm) which can be due to the aberrations induced by PDMS material in the detection path. Figure 4(d) shows the image of a passing bead along the axial direction. The intensity profile at the center of the bead, Fig. 4(e), demonstrates achievement of a 2.4-μm FWHM in the axial direction; this measurement is in good agreement with the simulated light-sheet thickness of 2.3  μm. The 3D experimental point spread function (PSF) of the SPIM system is visualized in Fig. 4(f).

### 3.2 *C. elegans SPIM Imaging with* the Add-On Optofluidic Device

**Fig. 5:**
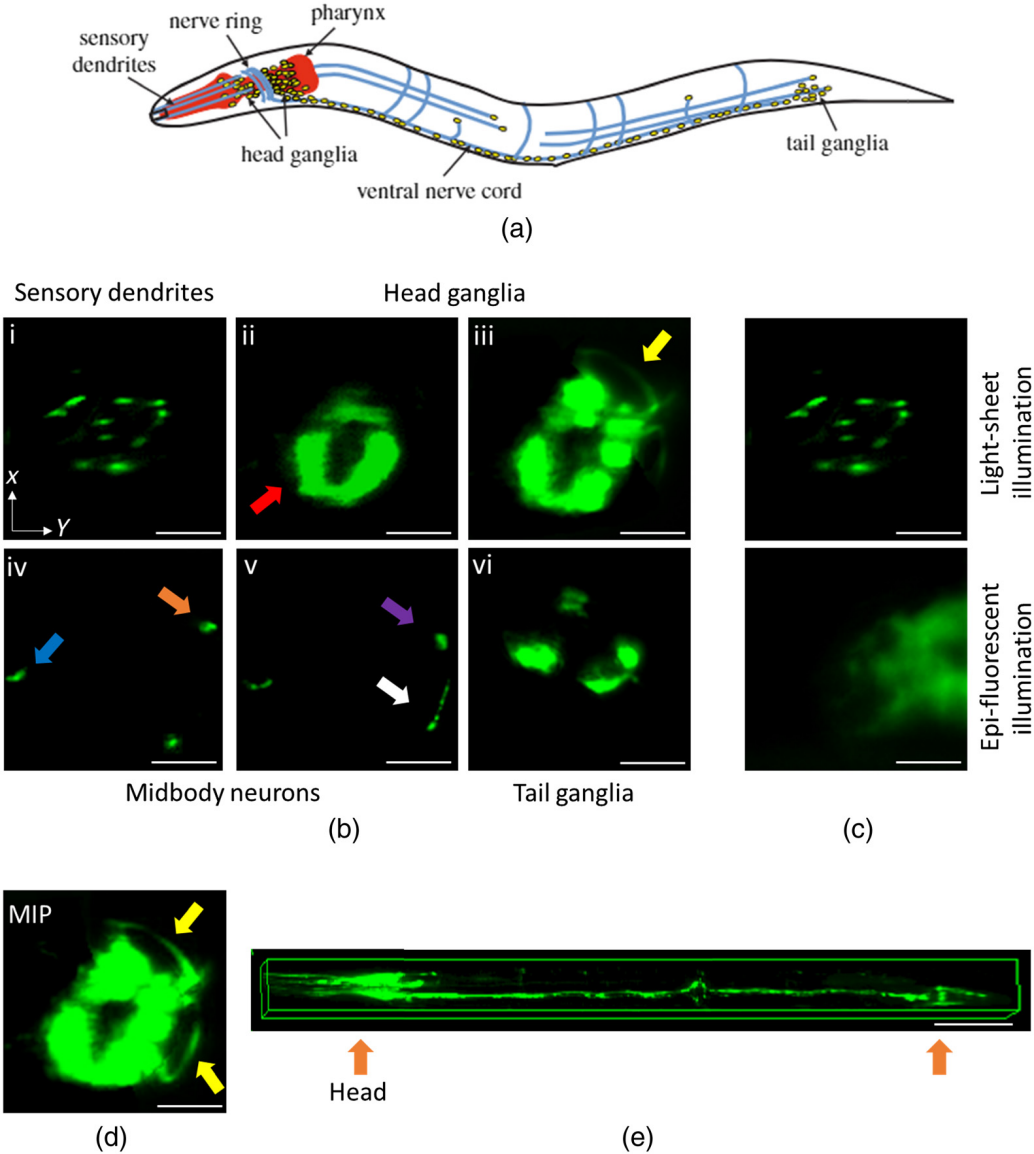
SPIM imaging of adult NW1229 *C. elegans* with the optofluidic device. (a) Overview of the nervous system of the *C. elegans*, reprinted with permission from Ref. 28. (b) Representative cross-sectional images of the pan-neuronally GFP expressing worm acquired with the optofluidic device under a conventional fluorescent microscope: (i) Cross-sectional image of the sensory dendrites; (ii), (iii) neurons in the brain of the *C. elegans.* The nerve ring is shown with a red arrow in (ii), and commissures are shown with yellow arrows in (iii); (iv), (v) cross-sectional images of the midbody section in which, orange and blue arrows indicate the ventral and dorsal nerve cords, respectively. Purple arrow shows the ventral nerve cord motor neurons and white arrow corresponds to the body commissure; (vi) cross-sectional image of the tail ganglia. (c) Comparing the contrast of the inverted microscope when operating in the epifluorescence mode (bottom) and the proposed light-sheet mode (top). Contrast enhancement is notable as the dendrites are only identified in the image acquired with the low-cost add-on light-sheet device. (d) The MIP of the acquired frames in the head region showing the nerve ring and head commissures. Scale bars in (b), (c), and (d) correspond to 20  μm. (e) Volumetric reconstruction of the worm. This 3D image was produced by stacking ∼1500 2D light-sheet frames (100  μm scale bar).

To investigate the feasibility of performing SPIM imaging with the low-cost add-on device, two *C. elegans* strains were imaged. Figure 5 shows the SPIM images obtained from an NW1229 *C. elegans* with pan-neuronal GFP expression. In this experiment, a gravid adult worm was loaded into the device at the flow velocity of $90\;{\mu m\cdot s}^{-1}$ and was continuously imaged during the ∼10-s period that took for the entire worm to pass through the stationary light sheet. The key characteristic of the NW1229 *C. elegans* is the clustering of neurons in the head and tail, connected across through a ventral cord, as shown in the Fig. 5(a).^28^^,^^29^ Figure 5(b) shows representative cross-sectional SPIM images of NW1229 neurons at different locations along the length of the worm. Characteristic fluorescent expressions of NW1229 can be clearly resolved. For example, sensory dendrites, head neuronal ganglia, ventral and dorsal nerve cords, and tail neurons are visualized with high contrast and resolution using the developed low-cost platform. Figure 5(bi) shows several fluorescent expressions extending from the brain to the tip of the nose. These expressions are consistent with those of the dendrites of the sensory neurons, which are of significant value to the disease models targeting neurodegenerative diseases such as Huntington’s disease.^30^ The acquired image of the same area in the head with the epi-illumination of the host inverted microscope is compared with those of the low-cost SPIM add-on in Fig. 5(c); the significant enhancement of image contrast can clearly be recognized in the image obtained with the low-cost add-on. This improvement is attributed to the elimination of out-of-focus fluorescent light of head ganglia region due to the selective planar excitation with the light-sheet. The contrast obtained from the dendrites of the sensory neurons with the low-cost add-on is comparable with images previously reported using conventional SPIM systems.^10^^,^^11^^,^^20^ Figure 5(bii-iii) shows the characteristic clustering of neurons inside the brain. The dense neuronal regions of head and tail ganglia^29^ express high fluorescent intensity which frequently saturate the acquired images. In the mid-body, Fig. 5(biv-v), the ventral and the dorsal nerve cords (orange and blue arrows, respectively), the ventral cord motor neurons (purple arrow), and the body commissures (white arrow) are clearly resolved. As expected, these neurons are mostly distributed close to the circumference of the worm cross section.^29^ Visualization of the ventral cord neurons along the worm body with high contrast and at high resolution is specifically useful for studies targeting neurodegenerative disorders such as Alzheimer’s disease and Parkinson’s disease.^31^^,^^32^ Lastly, neurons in the tail ganglia are visualized in Fig. 5(bvi), highlighting the most distal part of the worms’ neuronal system. Figure 5(d) shows the maximum intensity projection (MIP) of the frames acquired from the brain, showing the commissures (yellow arrows).^33^ The real-time image sequence of the adult worm expressing GFP pan-neuronally is shown in Video S1 in the Supplementary Material. Volumetric visualization of the worm’s entire neuronal system could be achieved by stacking the ∼1500 2D SPIM cross-sectional images as shown in Fig. 5(e). The results presented in Fig. 5 confirmed the ability of the developed low-cost optofluidic platform in visualizing the neuronal system of the worm with high contrast and resolution. Possibility of rendering the volumetric fluorescent expression of the entire worm for studying the neuronal processes in their natural 3D milieu has also been demonstrated.

**Fig. 6:**
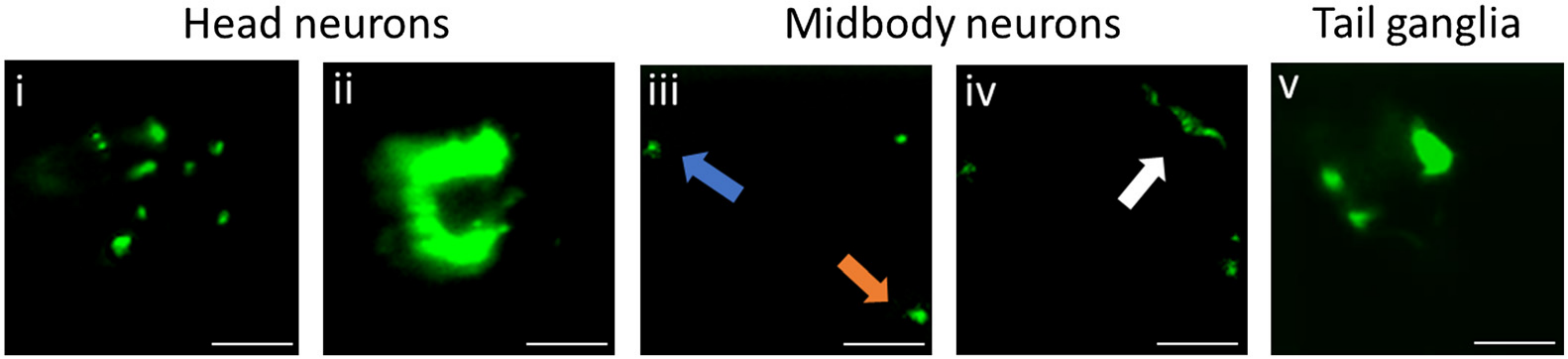
Fast cross-sectional image acquisition of *C. elegans* NW1229 strain at $450\;\mu m\cdot s^{-1}$ velocity using the add-on optofluidic device. Fluorescent expressions in (i) correspond to sensory dendrites. Axon bundles in head ganglia is observed in (ii). (iii), (iv) The neuronal features in the midbody region, such as ventral nerve cord (orange arrow), dorsal nerve cord (blue arrow), and the commissure (white arrow). Tail neurons are shown in (v). Scale bars correspond to 20  μm.

To explore the possibility of improving the imaging speed, an NW1229 worm was imaged while flowing at a higher velocity of $450\;{\mu \cdot s}^{-1}$. Figures 6 and Video S2 in the Supplementary Material show the possibility of rapidly imaging the entire nervous system of the worm without compromising the resolution. At such a flow velocity, the entire worm was imaged in ∼2  s with ∼600 acquired cross-sectional images, which ensured Nyquist sampling. For example, Fig. 6(i) shows fluorescent expressions associated with dendrites of sensory neurons, which is very comparable to those captured at the lower velocity in Fig. 5(bi). Similarly, the ventral nerve cord (orange arrow), dorsal nerve cord (blue arrow), and midbody commissure (white arrow) in worm’s midbody region are resolved with contrast and resolution comparable to those obtained at the low velocity, Fig. 6(iii-iv) versus Fig. 5(biv-v). These results confirm the ability of the optofluidic add-on device in rapid (∼2  s) imaging of *C. elegans* with high contrast and without the need for worm immobilization. This demonstration can open the door for downstream utilization of the developed low-cost optofluidic SPIM add-on for high-throughput imaging of *C. elegans*.

**Fig. 7:**
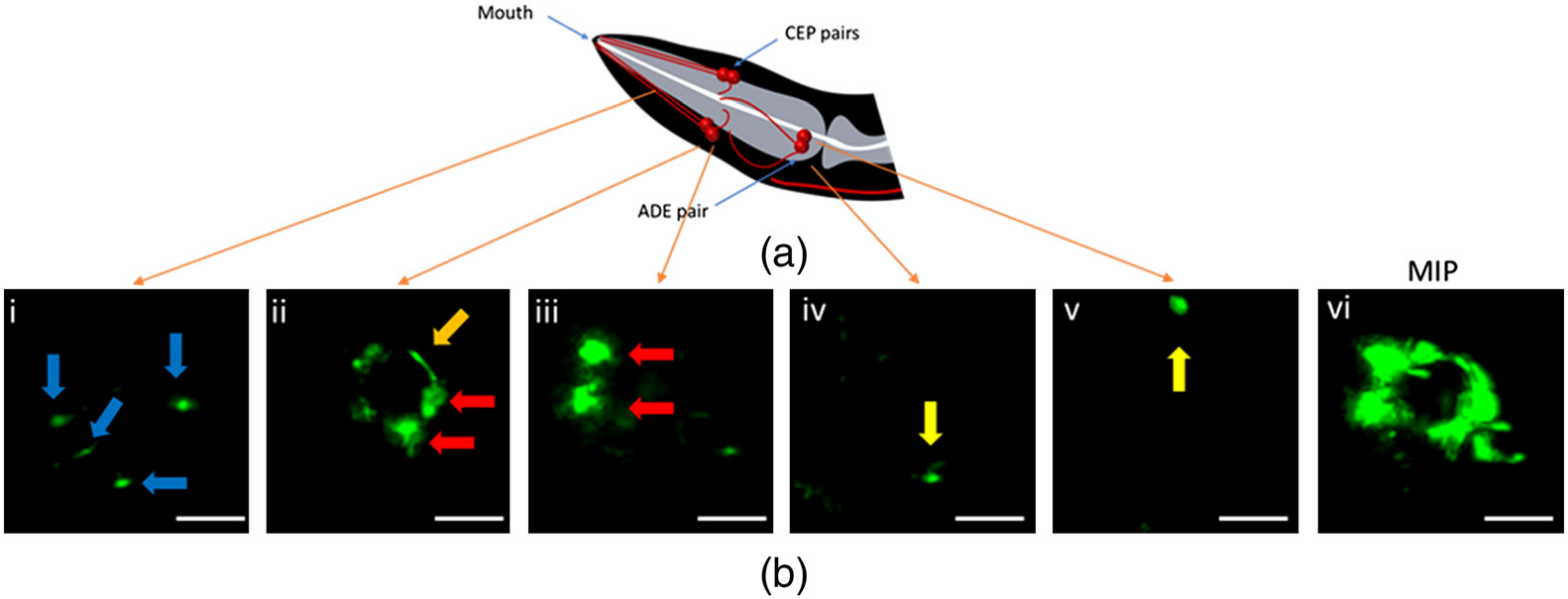
SPIM imaging of adult BZ555 *C. elegans* with the optofluidic device. (a) Schematic presentation of the head DNs. The fluorescent light-sheet images acquired with low-cost add-on device are shown in (b): (i) Cross-section image of the dendrites of the four CEP neurons; (ii), (iii) CEP neuron pairs (red arrows) and their axonal processes shaping the nerve ring (orange arrow); (iv), (v) ADE pair (yellow arrows); (vi) MIP image showing the two CEP pair and their axonal processes. Scale bars correspond to 20  μm.

Parkinson’s disease (PD) is a long-term neurodegenerative disorder characterized by the loss of DNs in the substantia nigra, causing severe motor and cognitive dysfunction. The BZ555 *C. elegans* strain, expressing GFP in its DNs [Fig. 7(a)],^34^ has been widely exploited as a model for studying PD. Figure 7(b) shows representative cross-sectional SPIM images of the anterior DNs of a BZ555 strain flowing at a velocity of $450\;\mu \cdot s^{-1}$ in the channel; here, the DNs and their axonal/dendritic processes are clearly resolved. Dendrites of the four CEP neurons (blue arrows) are shown in Fig. 7(bi). CEP neurons cell bodies (red arrows) along with the axons (orange arrow) are clearly visualized in Fig. 7(bii-iii). ADE neurons (yellow arrows) are shown with high contrast in Fig. 7(biv-v). Figure 7(bvi) shows a MIP image showing the two CEP neuron pairs and their axonal processes in a single image. Presented SPIM images of BZ555 *C. elegans* strain demonstrate the possibility of resolving a DN or a pair of DNs in a single frame which normally cannot be achieved with the host wide field microscope. The acquired real-time image sequence of the DNs of this worm strain is presented in Video S3 in the Supplementary Material.

The experimental results of Figs. 5, 6, and 7 confirm the ability of developed low-cost solution in rapidly acquiring high-contrast SPIM images of *C. elegans*. Given the fact that conventional SPIM systems are expensive and scarce, the developed platform has great potential to make significant contributions to the field of biology by offering a low-cost solution for converting the commonplace conventional wide-field microscopes to a fast SPIM system. The optical sectioning ability of the developed add-on enables volumetric visualization of individual neuronal features. This capability can open the door for studying the effects of environmental toxicants on specific neurons with a conventional microscope.^31^ Another significance of the developed low-cost solution is its ability to continuously image the cross sections of *C. elegans* as they pass through the stationary light sheet. This feature is specifically important in biological studies involving whole-body interrogation of large populations of *C. elegans*. Currently, most of the *C. elegans* studies performed on-chip require worm immobilization to obtain high-resolution fluorescent images, commonly in the top-view longitudinal orientation.^34^^,^^35^ The performance of these platforms is limited due to the intrinsic compromise between imaging speed and resolution as high NA objectives do not offer a large enough field of view to image the entire ∼1-mm long worm at once. The proposed low-cost solution, on the other hand, combines the planar illumination of the light-sheet microscopy with the continuous flow of the microfluidics platform, to enable rapid and high-contrast SPIM imaging of worm cross sections without the need for immobilization. Moreover, the acquired cross-sectional images can be stacked to generate 3D images of the worm without compromising the assay speed.

## 4 Limitations and Future Work

In this work, we demonstrated the possibility of low-cost SPIM imaging of an entire worm in ∼2  s; however, continuous high-throughput imaging of a population of worms for drug discovery or chemical screening applications requires modifications to the microfluidic design to enable smooth passing of the worms in the channel. For example, designing a microfluidic channel with smoother L-shape bend to ease the serial passing of many worms and development of image processing algorithms for compensating for discontinuous motion artifacts are modifications we are currently pursuing to address this limitation. Moreover, the fact that a cross-section of the worm is being imaged in the developed add-on can enable utilization of high NA lenses with smaller field of view for applications requiring better resolutions. We are currently changing the manufacturing protocol of the developed technology to examine such possibility.

## 5 Conclusion

We have designed and developed a low-cost add-on PDMS-based optofluidic chip for rapid and immobilization-free SPIM imaging of *C. elegans* with a conventional fluorescent microscope. The add-on device integrates the illumination path of the SPIM within the PDMS chip, enabling continuous imaging of the cross section of *C. elegans* as they pass through a microfluidic channel. The FWHM width of the PSF was measured as 1.1 and 2.4  μm in the lateral and axial directions, respectively. Experimental results from imaging NW1229 and BZ555 strains of *C. elegans* confirmed the ability of the system in producing optically sectioned and high contrast images of the entire neuronal system in few seconds. The possibility of generating volumetric images of *C. elegans* neuronal system from cross-sectional images was also examined. This work has great potential to make significant contributions to the field of biology by offering a low-cost solution for converting the commonplace conventional wide-field microscopes to an SPIM platform for studying neuronal expressions of *C. elegans*.
