# Supplementary material for: Low-cost optofluidic add-on enables rapid selective plane illumination microscopy of C. elegans with a conventional wide-field microscope
Source: J Biomed Opt. 2021 Dec 10;26(12):126501. doi: 10.1117/1.JBO.26.12.126501 (PMC8664272; doi:10.1117/1.JBO.26.12.126501)
Supplement: Supplementary file 1 [file JBO_026_126501_SD001.pdf]

Supplementary information:

Fabrication of the optofluidic device

To fabricate the optofluidic device, photolithography and soft lithography techniques were used to prepare a silicon (Si) wafer master mold and the PDMS negative replica, respectively. Briefly, the computer-aided design (CAD) Solidworks software (2021 SP2.0) was used to sketch the desired pattern and prepare it for printing. The photolithography mask was fabricated by printing the design on a 25000 DPI transparency sheet (CAD/Art Services Inc., USA). A Si wafer with 4-inch diameter and 500-550 µm thickness was surface treated for enhancing the photoresist adhesion using acetone, isopropanol, and DI water, followed by Oxygen plasma (PDC-001-HP Harrick Plasma, USA) for 30 s. Then, to fabricate the mold, four milliliters of SU-8 2075 photoresist (MicroChem Corporation, USA) was poured at the center of the Si wafer and coated using a spinner machine (Brewer Science® Cee® 200X, USA) at 3500 rpm for 30 s to obtain a 65 µm-thick layer. After soft-baking the wafer at 65˚C for 3 minutes and 95˚C for 6 minutes, the wafer was exposed to 365 nm light for 20 s (UV-KUB 2, KLOE, France) at a power density of 10 mW/cm^2. Then, the wafer was post-baked for 1 minute at 65 ˚C and for 4 minutes at 95 ˚C. Finally, the mold was rinsed for 2 minutes in SU-8 developer to remove the uncured SU8, leaving the final pattern for soft lithography.

To fabricate the optofluidic device, PDMS elastomer and curing agent (Dow Corning, USA) were mixed at 10:1 ratio, degasified, and poured on the above Si-SU8 master mold to make the negative replica. After curing for 2 hours at 80°C, the cured PDMS layer was oxygen plasma treated and bonded to another flat PDMS layer. Afterward, the device was cut from the side facet very close to the imaging region. Cutting the device caused roughness at the air-PDMS interface. To make the roughed facet optically flat, device was stamped from the cut side on a glass slide coated with a very thin layer of PDMS prepolymer. After being cured for 30 min at 70°C, the device was separated from the glass to achieve a roughness-free surface. To facilitate the vertical positioning of the add-on device on the inverted microscope stage, the device was finally bonded to a glass slide at a location away from the optical path.

C. elegans preparation

Synchronization: Gravid hermaphrodite adults were washed off the NGM plate using M9 buffer (3 g KH2PO4, 6 g Na2HPO4, 5 g NaCl, and 1 ml 1 M MgSO4 in 1 L distilled water) in a 15 ml Eppendorf tube and treated with a solution of 3.875 ml double-distilled water, 125 µL 1 M NaOH, and 1 ml commercial bleach for 10 minutes. Following the treatment, the eggs were collected by centrifuging the sample at 1500 rpm for two minutes and allowed to hatch to L1 larvae overnight in M9 buffer using a RotoFlex™ tube rotator (RK-04397-40, Cole-Parmer, Canada). The hatched larvae were seeded on top of freshly prepared NGM plates and collected on the day of the experiment for imaging.

Video S1: SPIM imaging of pan-neuronal GFP-expressing NW1229 c. elegans strain in 10 seconds.

Video S2: Fast SPIM imaging of pan-neuronal GFP-expressing NW1229 c. eleganstrain in 2 seconds.

Video S3: Fast SPIM imaging of bz555 c. elegans strain, expressing GFP in the dopaminergic neurons.
